# Surface Modification of Flax Fibers with TMCTS-Based PECVD for Improved Thermo-Mechanical Properties of PLA/Flax Fiber Composites

**DOI:** 10.3390/polym16030360

**Published:** 2024-01-29

**Authors:** Ghane Moradkhani, Jacopo Profili, Mathieu Robert, Gaétan Laroche, Saïd Elkoun, Frej Mighri

**Affiliations:** 1 Center for Innovation in Technological Eco-Design (CITE), University of Sherbrooke, Sherbrooke, QC J1K 2R1, Canadamathieu.robert2@usherbrooke.ca (M.R.); 2 Research Center for High Performance Polymer and Composite Systems, CREPEC, Montreal, QC H3A 0C3, Canada; 3 Quebec Center for Functional Materials, QCAM, Montreal, QC H2V 0B3, Canada; jacopo.profili@crchudequebec.ulaval.ca (J.P.);; 4 Centre de Recherche du Centre Hospitalier Universitaire de Québec, Hôpital St.-François d’Assise, Quebec, QC G1L 3L5, Canada; 5 Department of Chemical Engineering, Laval University, Quebec, QC G1V 0A6, Canada

**Keywords:** flax fiber, PECVD, organosilicon, mechanical properties

## Abstract

Significant progress has been made in recent years in the use of atmospheric pressure plasma techniques for surface modification. This research focused on the beneficial effects of these processes on natural by-products, specifically those involving natural fiber-based materials. The study explored the deposition of hydrophobic organosilicon-like thin films onto flax fibres through plasma-enhanced chemical vapour deposition (PECVD), using tetramethylcyclotetrasiloxane (TMCTS) as the precursor. After the successful deposition of hydrophobic organosilicon-like thin films onto the flax fibres, polylactic acid (PLA) composite materials were fabricated. This fabrication process sets the stage for an in-depth analysis of the modified materials. Subsequently, these flax fabrics were subjected to meticulous characterization through scanning electron microscopy (SEM), Fourier-transform infrared spectroscopy (FTIR), X-ray photoelectron spectroscopy (XPS), and contact angle measurements. The results demonstrated successful TMCTS deposition on the surface which led to a complete hydrophobization of the flax fibers. Mechanical tests of the PLA/flax fibre composites revealed a significant improvement in load transfer and interfacial compatibility following the surface modification of the flax fibres. This improvement was attributed to the enhanced adhesion between the modified fibres and the PLA matrix. The findings highlight the potential of TMCTS-based PECVD as a practical surface modification technique, effectively enhancing the mechanical properties of PLA/flax fibre composites. These developments open exciting possibilities for sustainable and high-performance composite materials in various industries.

## 1. Introduction

Natural fibre composites have gained attention in both academic and commercial communities due to their appealing combination of low density, eco-friendliness, and mechanical performance [1,2]. Flax fibre, a member of the best family, is already used as a reinforcement for composite materials [3]. Its high cellulose content, excellent tensile strength, flexibility, and elasticity make it an attractive choice for different sectors [4,5]. However, despite extensive research in the past two decades, the inadequate interfacial adhesion between flax fibres and polymer matrices remains a major challenge, limiting the wide employment of these materials in composite materials [6,7,8]. Moisture absorption in composite materials reinforced with flax fibres has also emerged as a significant concern, impacting the structural integrity and performance of these composites over time [9,10]. To address these challenges and ensure the long-term durability of natural fibre composites, effective strategies for managing moisture are necessary [11,12]. One promising approach is the application of barrier coatings on natural fibre substrates. These coatings enhance the performance and functionality of natural fibres, particularly in environments characterized by high humidity or aggressive conditions. Various techniques, including deep coating, sol-gel deposition, plasma treatment in combination with chemical vapour deposition, and electro-spinning, have been explored for surface modification of fibres [13,14,15,16]. These methods enable the development of protective layers that improve the robustness and durability of natural fibres [17]. For instance, in a study conducted by Liu et al. [18], a wet-chemical approach was employed to enhance the water resistance of flax fibre composites. The researchers create the composites by combining dried distiller grains with soluble (DDGS) and flax. They then applied a coating of acrylate epoxidized soybean oil (AESO) to the composites and initiated a polymerization process using UV light (λ < 325 nm). Remarkably, the coated DDGS-flax composites exhibited a significant reduction in water uptake compared to their untreated counterparts. Surprisingly, this technique had minimal impact on the colour and mechanical properties of the composites while subtly influencing the surface roughness. In another innovative study, Ishak et al. [19] directly addressed the challenge of poor interfacial adhesion in flax fibre composites. By employing a dip-coating method to functionalize flax fibres with 1,3-divinyltetramethyldisilazane (DVTS), they significantly improved compatibility with silicone rubber matrices. This advancement led to an increase in stiffness, with Young’s modulus of the DVTS-functionalized composites reaching approximately 3.6 ± 0.5 MPa, marking a 40% improvement over non-functionalized counterparts, showcasing the effectiveness of the DVTS functionalization in strengthening the mechanical properties of silicone-based composites.

In the last years, plasma modification has also emerged as an alternative technique for enhancing the surface properties of natural fibres while preserving their internal components. This method offers a promising solution to create barrier coatings on flax fibres by changing the surface functionalities [20,21]. Compared to the other conventional methods, plasma-based surface treatments exhibit several advantages such as low process temperatures, cost-effectiveness, and environmental safety. In addition, this process induces non-conventional modifications on the surface, such as dry-etching capitalizing on the creation of free radicals in the gas phase [21,22]. These alternative chemical processes strongly enhance the physical adhesion of surfaces when in contact with adhesive materials.

More recently, some studies have explored the use of atmospheric-pressure plasma treatments to modify natural fibres by incorporating precursor materials [23,24]. For instance, Samanta et al. [25]. utilized atmospheric pressure glow plasma with a combination of helium and 1,3-butadiene to modify woven cellulose textiles. The plasma treatment introduced functional groups containing CH_x_, such as CH_2_ and CH_3_, effectively resulting in a durable and long-lasting hydrophobicity on the textile surfaces. In the study conducted by Özdoğan et al., the effectiveness of surface modification treatments on the mechanical properties of flax polypropylene composites was explored. Specifically, they used atmospheric plasma treatment with argon gas at 240 W for 120 s on flax/polypropylene nonwoven mats. This treatment significantly enhanced the composite’s mechanical strength, increasing the tensile and flexural modulus by 58% and 70%, respectively. Notably, when compared to alkaline-treated mats (using a 4% NaOH solution), the plasma treatment under argon gas resulted in a further 16% improvement in the flexural modulus. This finding underscores the potential of atmospheric argon plasma treatment as a superior method for augmenting the mechanical properties of natural fibre-reinforced thermoplastic composites. Similarly, Özdoğan et al. [26]. demonstrated that atmospheric plasma treatment with argon gas at 240 W for 120 s on flax/polypropylene nonwoven mats significantly enhanced their mechanical strength. This treatment increased the tensile and flexural modulus by 58% and 70%, respectively, and even outperformed alkaline treatments with a 4% NaOH solution, particularly in flexural modulus.

Taking a different approach, Téraube et al. [27]. employed the PECVD technique to enhance the compatibility of flax fibres with dispersible polymers. Through the introduction of tetrafluoromethane (CF_4_) and pentafluoroethane (C_2_F_5_H) mixed with argon gases, the researchers successfully generated free radicals on the surface, enabling the covalent grafting of fluorine atoms on the fibres. This approach reduced the polarity of the surfaces, improving wettability without compromising mechanical properties. It is interesting to note that the innovative use of greenhouse gases and refrigerants as plasma precursors holds promise for the development of advanced materials from by-products.

Although the development of the plasma process at atmospheric pressure seems promising, additional studies are necessary to better understand the physio-chemical processes induced during the plasma-surface interactions on natural fabric. Therefore, the main objective of this research is to study the fragmentation of organosilicon precursors (i.e., 2,4,6,8-Tetramethylcyclotetrasiloxane or TMCTS) on natural flax fibres to enhance their mechanical properties in composites. In this work, the obtained results will help clarify the long-standing challenge of inadequate interfacial adhesion between flax fibres and polymer matrices in flax fibre composites.

The obtained results highlight the successful deposition of water-repellent coatings on both sides of the substrate layer using non-thermal plasma at atmospheric pressure. Characterization techniques, including SEM, FTIR, and contact angle measurements, have been used to correlate the mechanical properties evaluated through DMA and DSC with the physicochemical characteristics of the surfaces. The study contributes valuable insights into enhancing the durability and moisture resistance of flax fibre composites.

## 2. Experimental

### 2.1. Materials and Method

Flax fabric in a unidirectional pattern, sourced from Fibers Recherches Développement (FRD),Troyes, France, displayed an average fibre diameter of approximately 0.22 mm, as determined through optical microscopy. It has a measured thickness of approximately 0.4 to 0.5 mm and a density ranging around 1.38 g/cm^3^. The sample size employed for plasma treatment measured 6 × 4 cm^2^. GENEQ Inc, Anjou, QC, Canada, provided the PLA 4032D sheets, while 2,4,6,8-Tetramethylcyclotetrasiloxane (TMCTS) was bought from Sigma-Aldrich, Oakville, ON, Canada. The selection of TMCTS as the coating material is based on its exceptional advantages over other coatings. It possesses transparency, hardness, and compatibility with organic compounds, allowing for effective bonding with different substrates and enhancing mechanical performance and long-term durability [28]. Additionally, TMCTS is non-toxic, making it environmentally friendly, and its plasma polymers demonstrate desirable properties, including hardness, low gas permeability, and high chemical resistance [29].

### 2.2. Sample Preparation for Plasma-Modified Flax Fibers

**Figure 1:**
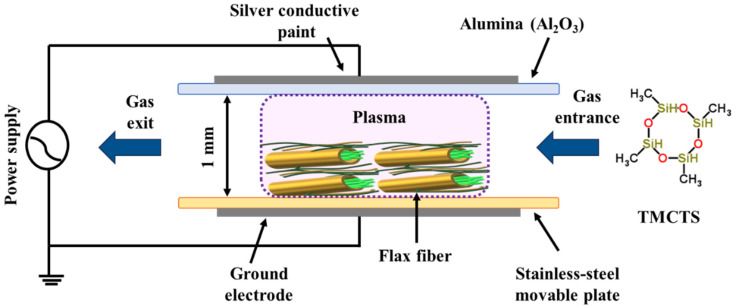
Schematic representation of the DBD reactor and TMCTS precursor used in the study.

The modified fibre preparation process used a setup illustrated in Figure 1. The setup consisted of a dielectric barrier discharge (DBD) reactor with two parallel plate electrodes. The upper electrode was a conductive paint-coated alumina sheet (5.0 × 3.0 cm, 0.64 mm thick), while the lower electrode was a stainless-steel plate (13 × 9 cm). To initiate the discharge, a sinusoidal voltage with a frequency of 4 kHz and a peak-to-peak amplitude of 18 kV was applied between the electrodes. The given conditions led to a discharge power of 0.25 W/cm^2^. The plasma treatment was conducted for 30 min with a 1 mm gap between the electrodes. For experimenting, the flax fabric substrate was firmly affixed to the lower electrode using Kapton tape. The tape was applied on all edges of the substrate. Subsequently, the chamber was evacuated to eliminate impurities and then filled with nitrogen gas to attain atmospheric pressure. The flow rates of nitrogen gas were accurately controlled using mass flow controllers from Bronkhorst, Rurlo, Netherlands. For controlled deposition, droplets of TMCTS (vaporized at 60 °C) were atomized (flow 0.03 mL/min) using a syringe pump from Fisher brand, Saint-Laurent, QC, Canada connected to the Mira Mist CE™ nebulizer (Burgener Research Inc., Mississauga, ON, Canada ) in nitrogen gas (3 L/min) between the electrodes. With this carefully designed experimental setup, the modified fibres were successfully prepared, ensuring precise control and accuracy throughout the entire process.

### 2.3. Composite Processing

To achieve moisture removal, the modified fibres underwent oven drying at 60 °C for 24 h before all treatments. For composite formation, a total of five unidirectional flax fabrics were combined with six layers of PLA, each PLA layer having a thickness of 0.025 mm. To ensure consistency, all flax fabrics were aligned in the same direction. The composite was created using thermocompression moulding. The optimal processing conditions involved sandwiching the samples between two Teflon sheets and applying a pressure of 44 MPa at a temperature of 180 °C for 5 min. As a result, the final thickness of the composite ranged between 2 mm and 2.20 mm, showcasing the integrated structure formed by the precise layering of flax fabric and PLA.

### 2.4. Characterization Methods

#### 2.4.1. Scanning Electron Microscopy (SEM)

The morphology of untreated and treated flax fibres was examined using an FEI Quanta250 SEM system from Thermo Fisher Scientific, Eugene, OR, USA. The SEM system operated at an acceleration voltage of 10 kV in secondary electron (SE) mode. To improve the imaging quality on low-conductivity flax fibre substrates, a fine layer of gold-palladium (Au-Pd) was coated onto all samples before imaging. This pre-treatment step ensured better visualization and enhanced the overall quality of the imaging results.

#### 2.4.2. Fourier-Transform Infrared Spectroscopy (FTIR)

FTIR spectroscopy was employed to analyze the chemical composition of both the pristine substrate surface and the surface of the plasma-modified substrate. For this analysis, an Agilent 600 instrument in attenuated total reflectance (ATR) mode was employed. To ensure accurate measurements, the samples were pressed onto an ATR crystal with a consistent force, resulting in a contact depth of a few microns. The measurements were conducted three times at three different locations on the sample, and the data were presented as means ± standard deviations, ensuring reliable and precise results. Each spectrum, obtained by averaging 128 scans, covered the range of 700–4000 cm^−1^ with a spectral resolution of 4 cm^−1^.

#### 2.4.3. X-ray Photoelectron Spectroscopy (XPS)

Fibre elemental composition was determined using XPS with a Kratos Axis Ultra DLD spectrometer. A power of 225 W and the monochromatized AlKα line (1486.6 eV) were used for sample excitation. To compensate for charging effects, a charge neutralizer was employed, and the samples were isolated on non-conductive adhesive tape. Data analysis utilized Casa XPS software (v2.3.18) with relative sensitivity factors (RSF) from Kratos Analytical. Three analyses per sample ensured chemical homogeneity and provided a reliable mean value and standard deviation.

#### 2.4.4. Differential Scanning Calorimetry (DSC)

The samples were analysed using DSC with a Q2000 TA Instruments Ltd, New Castle, DE, USA, in a nitrogen-controlled environment. The measurements involved a temperature range from 0 °C to 220 °C and included the recording of DSC curves in three stages: initial heating, cooling, and subsequent heating. To ensure the accuracy of the results, the data from the second heating stage were analysed in a way that minimised the impact on the thermal history of samples. The temperature increased at a rate of 10 °C per minute. This method provides a comprehensive comprehension of the thermal behaviour of the samples and enables precise characterization.

#### 2.4.5. Dynamic Mechanical Analysis (DMA)

The Q850 DMA system from TA Instruments Ltd. (New Castle, DE, USA) was utilized to perform comprehensive dynamic mechanical analysis on composite samples. The study aimed to explore the mechanical properties of materials as a function of temperature, ranging from −20 °C to 140 °C at a constant rate of 3 °C per minute. The frequency was maintained at 1 Hz throughout the experiment. The single cantilever bending method was employed to evaluate the mechanical properties, with all samples manufactured to exact dimensions of 2 × 12 × 17.5 mm for comparability.

## 3. Results and Discussion

### 3.1. Comparative Images of Flax Fibers Pre and Post Treatments

**Figure 2:**
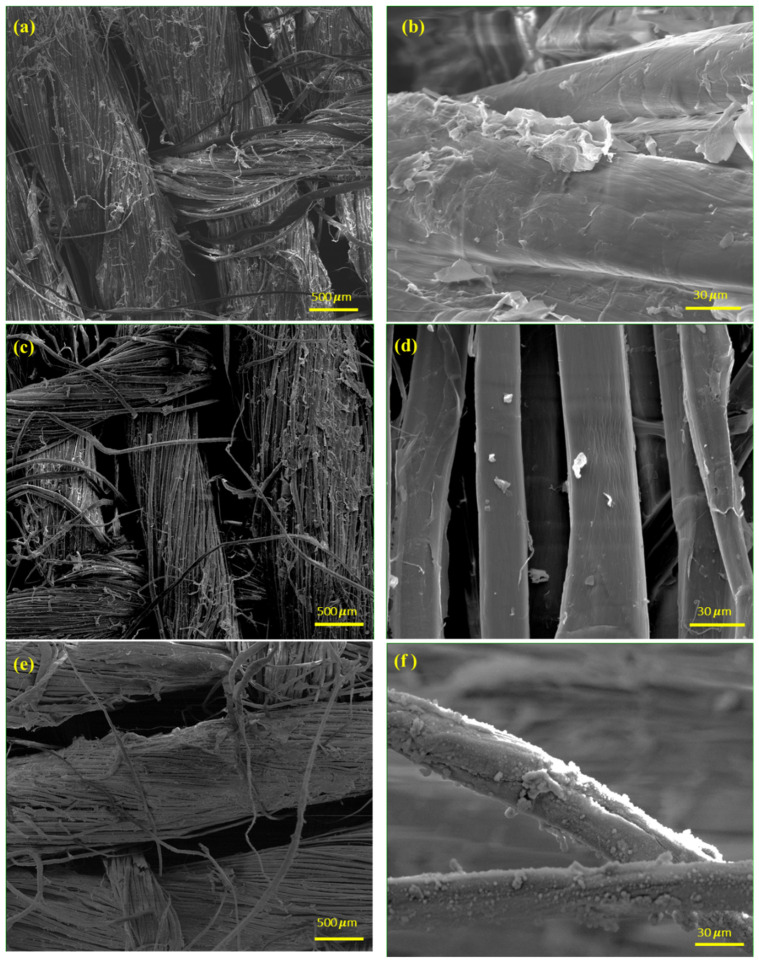
SEM Images of Flax Fiber Substrates—Untreated (**a**,**b**), Plasma-Treated with N_2_ Gas (**c**,**d**), and TMCTS-Coated after Plasma Treatment (**e**,**f**).

The SEM images of the flax fabrics before and after modification are depicted in Figure 2 at two different magnifications. Figure 2a,b clearly show the presence of small features on the surfaces. After plasma treatment with N_2_ gas (Figure 2c,d), noticeable changes in the surface texture become apparent, suggesting alterations in the physical properties of the substrate. The smoother appearance observed at high magnitude indicates that the plasma treatment has effectively modified the surface characteristics of the fibres. These findings align with our previous research [28] on the impact of plasma treatment on the removal of natural waxy substances from the fibres, indicating the treated state of the samples. Thus, the results reinforce the efficacy of plasma treatment to replace conventional wet methods to “clean” and/or functionalize the surface of natural fibres in dry conditions.

Further modifications were carried out using the N_2_/TMCTS treatment, leading to the deposition of a thick layer of organosilicon on the fibre surface (Figure 2e,f). The SEM images highlight the presence of a thin layer uniformly covering the entire surface of the fibres. This suggests a complete and homogeneous modification achieved through the process. These findings confirmed the morphology of the TMCTS-based coatings recently obtained with similar experimental conditions on natural materials [29]. More specifically, the authors employ a uniform and atmospheric-pressure dielectric barrier discharge (DBD) in helium combined with the TMCTS precursor to modify unrefined Kraft paper. Through high-resolution SEM imaging, they highlight the presence of micro and nanoscale fibrous structures from the paper. The authors suggest that these structures played a crucial role in influencing the heterogeneous growth dynamics of the organosilicon film. Similarly, Denes et al. [30] conducted a study using a cold plasma treatment technique utilizing oxygen gas to deposit “polymerized” hexamethyldisiloxane (P-HMDSO) onto wood surfaces. They observed that shorter treatment times did not result in complete surface coverage, but after just five minutes of exposure to the HMDSO-plasma environments, a uniform coating with a distinct nodule-like surface structure was achieved. Interestingly, longer plasma irradiation further enhanced the smoothness of the substrate surfaces. The SEM images presented in Figure 2 indicate that employing a uniform atmospheric-pressure DBD, along with TMCTS, results in a consistent and comprehensive organosilicon coating that uniformly covers the entire surface of the flax substrate. This SEM-based observation significantly contributes to the understanding of the effectiveness of this technique in achieving uniform organosilicon coatings on substrates.

### 3.2. FTIR of Non-Treated and Treated Flax Fiber

**Figure 3:**
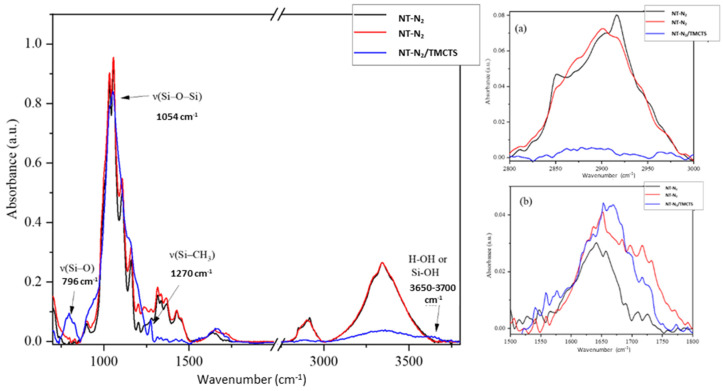
FTIR Spectrum of Flax Fiber Substrates—Untreated, Plasma-Treated with N_2_ Gas, and TMCTS-Coated after Plasma Treatment. Zoom (**a**) CH region, (**b**) carbonyl region.

Figure 3 depicts the chemical analysis from the ATR-FTIR spectroscopy. The results highlight the distinctive spectral characteristics of untreated flax fibre and two plasma-treated samples. Valuable insights into the chemical changes induced by N_2_ gas and N_2_/TMCTS plasma treatments can be obtained by comparing these spectra.

The presence of hydroxyl (OH) stretching absorption, indicating strong hydrogen bonding, is evident at 3340 cm^−1^. Pectin (waxes) can be associated with two distinct bands at 2915 and 2850 cm^−1^. A wavenumber of 1640 cm^−1^ corresponds to the vibrational mode of water molecules absorbed within the cellulose structure, while 1432 cm^−1^ is linked to the asymmetric deformation of δ(C–H_3_) in lignin [31,32]. The microcrystalline cellulose revealed three peaks at 1370 cm^−1^, 1335 cm^−1^, and 1315 cm^−1^. Polysaccharides in cellulose exhibit significant bands at 1160 cm^−1^ (ring breathing) and 1108 cm^−1^ (glycosidic ether band). The presence of amorphous cellulose in flax fibres is evidenced by distinctive C-O stretching peaks at 1056 and 1033 cm^−1^, alongside CH bending at 900 cm^−1^ [33,34]. Table 1 provides a summary of the assigned characteristic bands.

Upon subjecting the flax fibre to plasma treatment using N_2_ gas some transformations in the ATR-FTIR spectra were observed (zoom (a)). Remarkably, the characteristic peaks associated with wax at approximately 2915 cm^−1^ and 2850 cm^−1^, which correspond to the asymmetrical and symmetrical stretching of CH_2_ groups, were effectively eliminated in agreement with previous findings [28]. In addition, the emergence of a distinct peak around 1720 cm^−1^ signified the presence of a carbonyl group (zoom (b)). These spectral variations reflect the profound influence of plasma treatment on reshaping the chemical composition of the flax fibre surface. A noticeable distinction is observed in the spectrum of the plasma treated with TMCTS substrate compared to the untreated substrate. The precise attribution of the absorptions within the range of 1000 cm^−1^ to 1250 cm^−1^ remains a challenge due to the overlapping of Si-O-Si and C-O-C vibrations. As a result, the discussion about the chemical modification of the substrate after plasma treatment mainly centres on the region between 1250 cm^−1^ and 4000 cm^−1^. Notably, the spectral analysis of the deposited plasma-treated sample reveals a strong reduction for the O-H vibrations in the range of 3000–3800 cm^−1^. However, the spectrum also exhibits an interesting feature—a subtle peak between 3600 and 3700 cm^−1^, indicating the presence of Si-OH groups [35]. Unlike a sharp peak, this distinctive band suggests the formation of silicon hydroxide (Si-OH) functional groups resulting from plasma-induced reactions. The energetic plasma species, in conjunction with the TMCTS precursor, likely facilitated the absorption of oxygen from the surrounding environment. Consequently, Si-OH groups were formed, giving rise to the appearance of this characteristic peak in the FTIR spectrum. This variation strongly indicates that the chemical changes observed primarily originate from the presence of the organosilicon coating. This finding aligns with the research conducted by other researchers where they also observed the absence of O-H bands in their investigation of plasma-treated kraft lignin [29].

One notable feature of plasma-deposited coatings on flax fibre is the distinct and wider peak corresponding to the Si-O-Si bond in the FTIR spectrum of the plasma-polymer compared to the untreated flax fibre. This broadening can be attributed to the presence of a thick organosilicon layer which completely covers the cellulosic substrates and makes invisible the absorption associated with cellulose at 1033 cm^−1^. The Si-O-Si peak, typically observed between 1000–1250 cm^−1^, also provides valuable insights into the molecular structure and arrangement of the coatings. In atmospheric pressure glow discharges, bonds such as Si-C, Si-H, and C-H are more susceptible to fragmentation, while Si-O bonds remain relatively stable. As a result, the siloxane groups containing the Si-O-Si resulted in a stronger and wider vibration signal in this region. The FTIR spectra exhibit a distinct peak at 1270 cm^−1^, which indicates the presence of asymmetrical deformation in the methyl groups of Si (CH_3_)_x_. This finding supports the interpretation of the nearby peak at approximately 1360 cm^−1^, which represents the Si-CH_2_-Si wagging deformation. This wagging deformation is a characteristic feature commonly observed in the structural framework of polycarbosilane materials and organosilicon networks. Importantly, the absence of corresponding peaks at 1370 cm^−1^ (δ (CH)) and 1335, 1315 cm^−1^ (δ (CH_2_)) indicates a relatively lower abundance of these organic functionalities in the plasma-deposited coating. At this stage, it is also important to note that the intensity of the Si-CH_x_ bonds remains low compared to similar results from the literature [36,37]. This suggests a stronger oxidation of the organosilicon layer, probably related to the presence of residual oxidant gases in the porous fibres during the process. Indeed, Levasseur et al. already highlighted the possibility of affecting the oxidation state of the organosilicon layer when porous substrates outgas in discharges at atmospheric pressure [38]. These observations are confirmed by the lower amount of CH_x_ bonds already described around 2900 cm^−1^ as well as the presence of a strong peak for the Si-O bonds at 796 cm^−1^. Consequently, these observations suggest that the coating process utilizing TMCTS through plasma deposition techniques induces changes, resulting in the disappearance of the absorptions related to the flax fibres. The fragmentation of the precursors remains high. Because of the low intensities of the peaks related to the carbon moieties one can suggest that a small oxidation of the layer occurs during the process, probably caused by the oxygen molecules trapped into the fibers. FTIR spectrum of flax fibre substrates with modifications, is summarized in Table 1, which provides the characteristic band assignments.

### 3.3. Contact Angle

**Figure 4:**
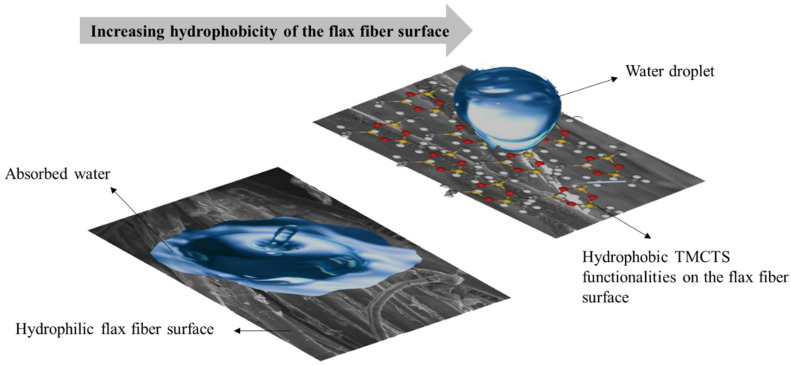
Schematic Illustration of TMCTS Deposition on Flax Fiber Surface during DBD Plasma Treatment in Nitrogen Process Gas and its Interaction with Water Droplets.

The impact of process gas containing TMCTS vapours on the water contact angle (WCA) of flax surfaces was investigated. Untreated flax fibres, which naturally have a high affinity for water, posed a challenge in accurately measuring the static water contact angle due to their significant water absorption upon droplet settlement. Conversely, flax samples treated with nitrogen exhibited comparable wettability to the untreated samples, indicating minimal changes in their wettability (schematic of the mechanisms highlighted in Figure 4). However, the introduction of TMCTS during the treatment process resulted in a remarkable increase in the contact angle measurement for the flax samples. The water contact angle reached approximately 139 degrees, indicating a substantial enhancement in hydrophobicity compared to both the untreated and nitrogen-treated samples. These findings were further substantiated by the low intensity of the absorptions in the O-H region for the deposited plasma-treated sample, as detected by FTIR spectroscopy. The absence of these vibrations confirmed the presence of an organosilicon coating on the flax surface, which contributed to the increased hydrophobicity. These findings are consistent with other research conducted in the literature [39]. This study investigated the stability of these coatings in various liquids, specifically food simulants. Interestingly, the presence of the coated paper samples remained stable even after 24 h of immersion and vigorous shaking in different food simulants, regardless of the specific chemical nature of the solution. Notably, the coated paper surface exhibited a highly hydrophobic character, with a static water contact angle value of 132 ± 8°. In a similar work, Babaei et al. [37], studied the water-repellency of cellulose filament (CF) films by depositing an organosilicon (SiOCH) thin film through DBD at atmospheric pressure. They successfully achieved a highly hydrophobic CF film while maintaining unchanged water vapour and oxygen transmission rates. Additionally, they explored the combination of SiOCH coating and carboxymethylcellulose (CMC) sealer, which significantly reduced water vapour and oxygen transmission rates, while also lowering water absorbency. These functionalization methods offer promising opportunities for developing sustainable packaging materials with tailored permeation properties. Accordingly, to these results, one can affirm that the variation in water droplet absorption observed during the treatment process be attributed to the integration of hydrophobic organosilicon functional groups onto the surface of flax fibres. In addition, the small oxidation occurring during the process and highlighted in the FTIR section does not strongly affect the hydrophobic proprieties usually observed in the literature with discharges created in the absence of oxidizing gases and with organosilicon precursors [40].

### 3.4. XPS Spectroscopy Examination: Investigating the Surface of Flax Fibers

XPS was employed for a precise analysis of the chemical composition of untreated and treated flax fibre substrates, providing valuable insights into the outermost surface layers of the deposited films. The data from Table 2 reveals that the untreated sample (NT) predominantly consists of carbon (75.12%) and oxygen (24.26%), with minor traces of nitrogen and silicon. In the plasma-treated sample with nitrogen gas (NT/N_2_), carbon (64.76%) and oxygen (31.26%) remain dominant, but nitrogen contents have notably increased due to the introduction of nitrogen gas during the treatment. This suggests successful surface modifications and functionalization of the flax fibres. The observation comes from the NT/N_2_-TMCTS samples exhibit stronger modifications: high silicon content (52%) and a significant reduction in carbon and oxygen. The observed atomic ratios strongly support the presence of a plasma-deposited thin film primarily composed of silicon, carbon, and oxygen. Notably, these atomic ratios closely align with those expected for the TMCTS precursor, further confirming the efficacy of TMCTS for successful surface modification. The [Si]/[C] ratio of 0.9 highlights the substantial silicon content in the plasma-deposited thin film, while the [O]/[Si] ratio of 2.29 indicates a higher proportion of oxygen relative to silicon. These ratios underscore the prevalence of Si-O-Si or SiO_2_ groups on the surface, emphasizing the successful incorporation of silicon in the plasma-modified flax fibre substrate. The results also confirm the observations from the FTIR analysis suggesting a low amount of Si-C_x_-Si bonds and a small intensity for the CH_x_ bonds.

**Figure 5:**
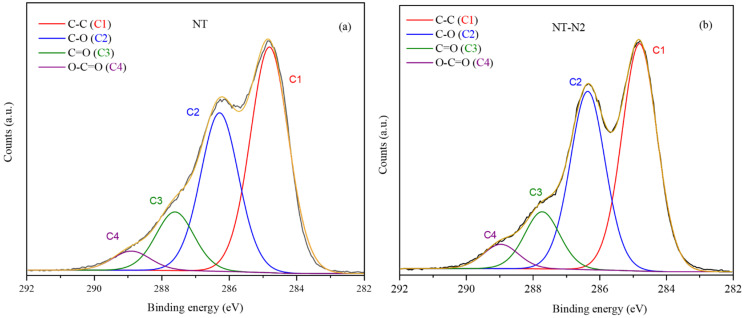

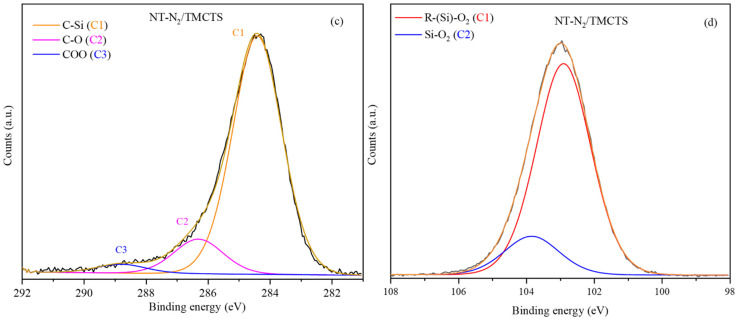
XPS spectra of (**a**) untreated fibre (NT), (**b**) plasma treated fibre with nitrogen (NT-N_2_), (**c**) TMCTS-Coated after fibre Plasma Treatment with N_2_ (NT-N_2_/TMCTS) and (**d**) TMCTS-Coated after fibre Plasma Treatment with N_2_ (NT-N_2_/TMCTS) and the presence of thin layer of Si-O-Si or SiO_2_ for C1s and Si 2p. Black and brown lines are the measured and fitted signals respectively.

To gain a deeper understanding of the chemical bonds present on the surface of the plasma-polymerized films, the high-resolution C1s peak was subjected to curve fitting analysis. The deconvoluted components in the C1s spectrum included C-C sp^3^ (284.8 eV), C-O (286.3 eV), C=O (287.6 eV), and O-C=O (288.8 eV), each with a full width at half maximum (FWHM) of 1.4 eV. The material showed the presence of C-C sp^3^, which was attributed to a combination of sp^2^ carbon originating from the aromatic cycles of lignin, as well as sp^3^ carbon (C-C/C-H) found in lignin, cellulose, hemicelluloses, and wax. The C-O peak in the spectrum indicated carbon atoms bonded to oxygen through a single bond, involving functional groups like COH, C-O-C, or phenyl-OH. Additionally, the C=O peak was associated with carbonyl carbon (C=O), while the O-C=O peak corresponded to sp^3^ carbon atoms present in ester and carboxyl groups. The untreated flax fibre showed a high carbon content, suggesting the presence of a hydrocarbon-rich waxy layer on its surface (Figure 5). In contrast, the plasma treatment with NT-N_2_ led to a slight increase in oxygen content and a consistent decrease in carbon content. This treatment also resulted in a higher proportion of carboxylic bond area, indicating not only the removal of the wax layer but also surface activation. FTIR results further supported these observations, confirming the effective removal of the waxy layer and surface activation by the plasma treatment. In Figure 5c, the deconvolution of the plasma-treated substrate was presented, with a specific focus on the C1s and Si 2p peaks. Notably, no changes were observed in the O1s peak following the plasma treatment. Analysis of the FTIR data provided valuable insights, suggesting that the carbon content predominantly consisted of Si-C bonds. As a result, the major contribution to the C1s peak was attributed to C-Si groups at 284.4 eV. Additionally, smaller contributions to the C1s peak were identified, including C-O sp^3^ (286.3 eV-C1) and O-C=O (288.8 eV-C2) components, primarily originating from the flax fibre substrate. This suggests that the heterogeneous surface topography of the flax fibres may lead to partial coverage after treatment, leaving some regions of the fibre substrate exposed. In Figure 5d, the high-resolution Si 2p spectrum was shown, commonly deconvoluted into four peaks based on the oxygen environment of the silicon atoms. An observed trend revealed that the Si 2p core peak shifted to higher binding energies with an increase in the oxygen environment of the bond. The film composition on the topmost layer was described as a thin polymer-like film primarily composed of Si-O-Si or SiO_2_ groups. The detection of SiO groups in the film, as revealed by FTIR analyses, indicates that the surface underwent oxidation, likely due to atmospheric oxygen or humidity post-plasma treatment. This oxidation, possibly enhanced by residual oxygen in the fibres, suggests a more complex surface chemistry than initially assumed, involving varied oxidation states. Such findings highlight the dynamic interaction between plasma modifications and environmental factors, warranting further study under controlled conditions for a clearer understanding of these processes.

### 3.5. Thermal Characterization of PLA/Flax Fiber Composites

The thermal properties of neat Polylactic Acid (PLA) and Flax/PLA biocomposites were examined using Differential Scanning Calorimetry (DSC). The DSC curves of the first and second heating cycles were analyzed to gain insights into the material behaviour. The obtained results are summarized below.

**Figure 6:**
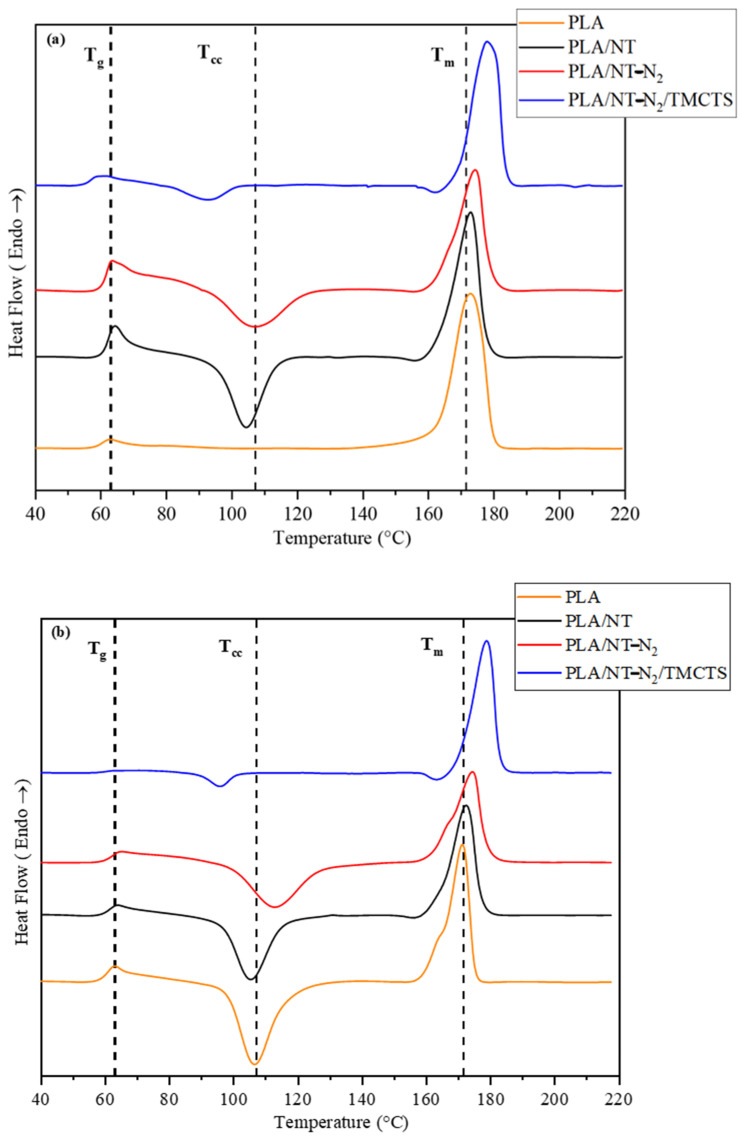
DSC Curves of PLA and PLA/Flax Fiber Composites during (**a**) First and (**b**) Second Heating, with a Heating Rate of 10 °C/min.

Figure 6 illustrates the DSC curve for the first heating cycle, revealing an endothermic peak within the range of 58–68 °C, corresponding to the glass transition temperature (T_g_). Interestingly, the PLA/NT-N_2_/TMCTS sample displayed a lower T_g_, indicating potential chemical interactions between the TMCTS coating and the PLA matrix. This interaction influenced the mobility of polymer chains, thereby enhancing the compatibility between the PLA matrix and flax fibres. The observed lower T_g_ suggested enhanced interfacial adhesion, which could promote more effective stress transfer between the two components.

The second peak observed in the DSC curves represents an exothermic cold crystallization process, signifying the transition from an amorphous solid to a crystalline solid. Neat PLA showed no crystallization throughout the heating process at a rate of 10 °C/min. However, the addition of flax fibres to the PLA/flax fibre composites formed cold crystallization peaks. This finding indicated an improvement in the crystalline properties of the PLA matrix, which could be attributed to the presence of flax fibres. This aligned with the findings stated in the reports, which confirmed that the inclusion of the additive improved the crystalline characteristics of PLA [41,42,43].

The DSC curves showed a lower cold crystallization temperature (Tcc) in the TMCTS-coated sample, suggesting the TMCTS coating acted as a nucleating agent, promoting the early formation of crystalline regions in the PLA matrix at lower temperatures, rather than hindering the crystallization process. This aligned with a study by Kassem et al. [44] investigated the DSC behaviour of SiO_2_-coated PLA composites, revealing a lower shift in T_cc_, indicating the nucleating effect of the SiO_2_ particles on PLA crystallization. The presence of SiO_2_ nanoparticles offered insights into their potential to enhance the crystalline properties of PLA matrices.

The third endothermic peak held vital significance in processing flax fibre composites, indicating the temperature to avoid flax fibre degradation. Remarkably, DSC results revealed higher melting points in PLA/NT-N_2_/plasma and PLA/NT-N_2_/TMCTS samples. Plasma treatment effectively removes impurities and defects from the natural fibres, promoting orderly polymer chain packing during melting. TMCTS coating enhanced fibre thermal stability, preventing degradation at elevated temperatures. These factors contributed to the elevated melting points in the respective composites.

In conclusion, the DSC analysis provided valuable insights into the thermal behaviour of neat PLA and flax/PLA biocomposites. The incorporation of TMCTS coating appeared to have the potential to enhance compatibility between the PLA matrix and flax fibres, thereby leading to improved interfacial adhesion and thermal stability.

Table 3 summarizes the DSC parameters for both PLA and PLA/flax fibre composites. These parameters offer important information about the thermal characteristics of the materials across the initial and secondary heating cycles.

### 3.6. Thermal Mechanical Analysis of PLA/Flax Fiber Composites

**Figure 7:**
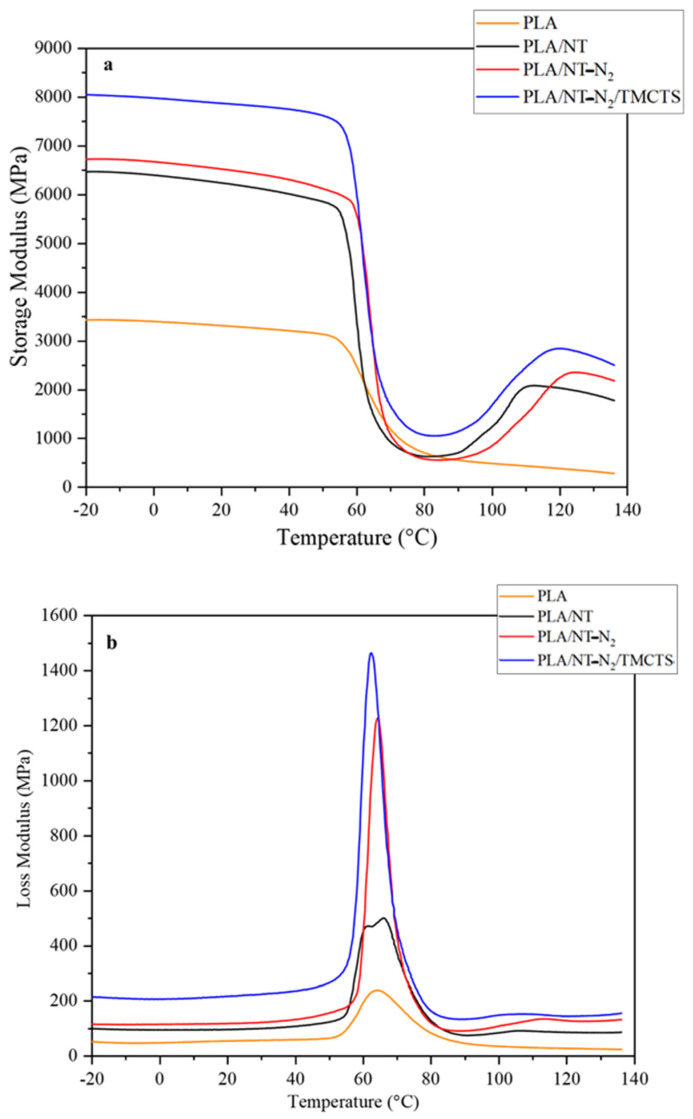
Variations in storage modulus (**a**) and loss modulus (**b**) of flax/PLA composites are explored under different temperatures, comparing treated and untreated samples.

The influence of temperature on the dynamic storage and loss modulus of PLA and PLA-based composites is depicted in Figure 7, where DMA measurements were conducted to assess the impact of elevated temperatures on the stiffness of composites. The storage modulus, a critical parameter reflecting a load-bearing capacity of material, was analyzed. Figure 7a highlights that PLA-based composites exhibit a greater storage modulus compared to the PLA matrix. This improvement can be attributed to the increased rigidity of the reinforcing fibres, resulting in a more efficient transfer of tension from the matrix to the fibres. Furthermore, the data presented in Figure 7a reveals a modest increase in the storage modulus of the PLA/NT-N_2_ composite compared to the PLA/NT composite. This observation suggests a slight improvement in the adhesion between the PLA matrix and the flax fibres following plasma pre-treatment. The removal of wax from the flax fibres seems to contribute to the enhancement of interfacial bonding.

In contrast, the PLA/flax composites coated with TMCTS exhibit a significant increase in storage modulus compared to other composites. This remarkable enhancement is attributed to a reactive coating that forms strong covalent bonds at the fibre-matrix interface, effectively enhancing load transfer and interfacial compatibility. The presence of TMCTS as a coating material not only improves the interfacial adhesion but also acts as a moisture barrier, preserving the mechanical properties of the flax fibres.

The notable increase in storage modulus observed in the PLA/flax composites coated with TMCTS demonstrates the immense potential of this coating technique in enhancing the mechanical performance of the composites. The combination of improved interfacial adhesion and moisture resistance positions these composites as highly suitable for load-bearing applications.

Remarkably, all flax/PLA composites exhibit a decrease in storage modulus around the glass transition temperature (Tg), followed by a subsequent increase above 80 °C, attributed to cold crystallization. This phenomenon aligns with prior research [28] where flax fibre was employed as a nucleator to augment the crystallization capability of PLA composites.

The loss modulus data for PLA composites is revealed in Figure 7b. Pure PLA exhibits the lowest loss modulus, consistent with the expected behaviour of a non-reinforced base polymer. By contrast, the PLA/NT composite shows an improved loss modulus, likely due to enhanced interaction between the PLA matrix and flax fibre reinforcements. Further increases in loss modulus are observed with the PLA/NT/N_2_ composite, indicating a boost in damping properties due to more effective molecular interactions. The most significant enhancement is found in the PLA/NT/N_2_-TMCTS composite, which consistently displays the highest loss modulus across all temperatures. This suggests that the TMCTS coating, in conjunction with plasma treatment, greatly improves the energy dissipation capability of the composite, making it particularly suitable for applications demanding high damping, such as vibration reduction or impact resistance. Shen et al. [45] studied on the dynamic mechanical properties of flax fibre-reinforced composites lends support to these findings. Their work confirms that stronger interfacial bonding, achieved through specific treatments and reinforcements, enhances both the stiffness and energy dissipation capabilities of composites. This enhancement is attributed to improved viscoelastic properties and a more effective interfacial phase that acts as a bridge within the composite material. Under dynamic loading, these composites show increased internal friction and shear strain mechanisms, leading to enhanced energy dissipation. Our observations in the PLA/NT/N_2_ and PLA/NT/N_2_-TMCTS samples, which show elevated storage and loss modulus, align with these findings, indicating a synergistic enhancement in both rigidity and damping characteristics due to the applied treatments.

**Figure 8:**
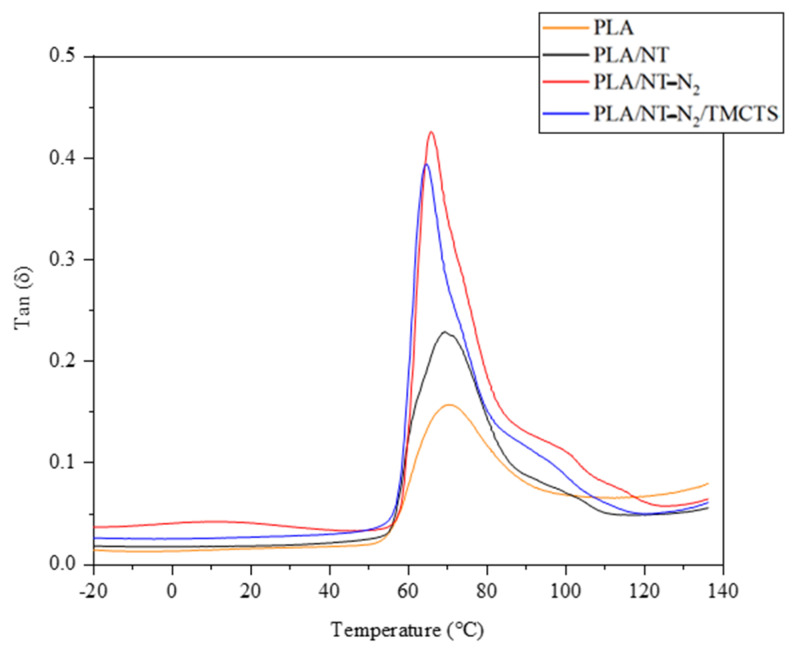
Variations in the loss factor of flax/PLA composites are explored under different temperatures, comparing treated and untreated samples.

PLA/NT-N_2_ exhibits the highest value of the Tanδ curve, as anticipated, owing to its limited molecular motion at higher temperatures (Figure 8). This restricted motion is attributed to the presence of NT-N_2_, which acts as a hindrance and impedes the movement of the polymer chains. Consequently, the higher Tanδ values observed for PLA/NT-N_2_ and PLA/NT-N_2_/TMCTS indicate superior damping characteristics compared to the PLA/NT and PLA composites. The lowest value of Tanδ is observed for the pure PLA composite, primarily due to its strong interfacial adhesion between the polymer matrix and the reinforcement. The interfacial adhesion facilitates efficient load transfer between the matrix and the reinforcement, resulting in enhanced load-bearing capacity. The strong adhesion restricts molecular mobility and reduces the ability of the polymer chains to dissipate energy, leading to a lower Tanδ value. It is worth noting that the composites exhibit a shift in Tg towards lower temperatures, indicating decreased thermal stability compared to the pure PLA composite. This phenomenon can be attributed to the presence of reinforcing elements in the composites, which may introduce defects, disrupt the polymer chain arrangement, and weaken intermolecular forces. Consequently, the composite materials display a reduced resistance to thermal degradation, as evidenced by the shifting of Tg to lower temperatures.

Table 4 summarizes the key dynamic mechanical properties of PLA and its composites. The data illustrates significant enhancements in mechanical properties with the incorporation of NT, pre-treatment, and TMCTS treatments in PLA composites.

## 4. Conclusions

In conclusion, this research successfully utilized atmospheric pressure plasma processing techniques, particularly PECVD with TMCTS as the precursor, to modify flax fibres. The deposition of hydrophobic organosilicon-like thin films on flax fibres led to enhanced mechanical properties and improved bond strength in Polylactic Acid (PLA)/flax fibre composites.

Characterization techniques confirmed the effectiveness of the plasma treatment. SEM observations demonstrated uniform and complete organosilicon coatings on the flax substrate. FTIR spectra showed the presence of the organosilicon coating, while XPS analysis supported successful surface modification with a plasma-deposited thin film primarily composed of silicon, carbon, and oxygen.

The hydrophobicity of the flax samples significantly increased due to the integration of hydrophobic organosilicon functional groups, as evidenced by the notable increase in water contact angle. The surface modification process using TMCTS through plasma deposition techniques effectively removed the waxy layer and activated the surface, enhancing the adhesion between the flax fibres and the PLA matrix.

Thermal analysis using DSC revealed that the TMCTS coating acted as a nucleating agent, promoting the early formation of crystalline regions in the PLA matrix, thereby improving the composite’s crystalline properties. Additionally, the coating enhanced the thermal stability of the fibres, preventing degradation at elevated temperatures.

Mechanical tests, DMA, demonstrated a significant improvement in load transfer and interfacial compatibility in the PLA/flax composites coated with TMCTS. The reactive coating formed strong covalent bonds at the fibre-matrix interface, resulting in enhanced load-bearing capacity and improved mechanical performance.

Overall, this research highlights the potential of atmospheric pressure plasma processing techniques to enhance the surface properties of natural fibres while preserving their internal components. TMCTS proved to be a promising precursor with exceptional advantages, including hydrophobicity, compatibility with organic compounds, and non-toxicity. The study offers valuable insights into creating barrier coatings on flax fibres, providing improved functionality and suitability for diverse load-bearing applications.

## Figures and Tables

**Table 1:** FTIR Peaks for Flax Fiber Substrates—Untreated, Plasma-Treated with N_2_ Gas, and TMCTS-Coated after Plasma Treatment.

Peak Location (cm^−1^)	Bond	Component
3650–3700	Si-OH	Isolated silanol
3344	ν (OH)	Hydrogen bonds
2915, 2850	ν (CH_2_)	Waxes
1640	δ (OH)	Adsorbed water within the fibre
1432	δ (CH_3_)	Aromatic lignin
1370	δ (CH)	Cellulose
1335, 1315	δ (CH_2_)	Cellulose
1270	ν (Si–CH_3_)	Silicon methyl group
1160	ν (C–C)	Polysaccharides in cellulose
1108	ν (C–O–C)	Polysaccharides in cellulose
1056, 1033	ν (C–OH)	Cellulose
900	ν (C–O–C)	Cellulose
796	ν (Si–O)	Silicone

**Table 2:** Atomic Composition and Relative Atomic Percentages of Flax Fiber Samples—NT (Untreated), NT/N_2_ (Plasma-Treated with Nitrogen), and NT/N_2_-TMCTS (Plasma-Treated with Nitrogen and TMCTS).

Sample Name	Atomic Percentage				Relative Atomic%	
	C	O	N	Si	[Si]/[C]	[O]/[Si]
NT	75.12	24.26	0.36	0.25	0	97.04
NT/N_2_	64.76	31.26	1.74	2.23	0.03	14.01
NT/N_2_-TMCTS	25.29	52	0	22.71	0.9	2.29

**Table 3:** DSC parameters of PLA and PLA/flax fibre composites.

Name	First Heating				Second Heating			
	T_g_ (°C)	T_cc_ (°C)	T_m_ (°C)	X_C_ (%)	T_g_ (°C)	T_cc_ (°C)	T_m_ (°C)	X_C_ (%)
PLA	63	-	173	57.81	63.1	106.6	171.4	48.41
PLA/NT	64.3	104.4	173.2	76.35	63.1	106.7	171.5	78.75
PLA/NT-N_2_	63.7	107.0	174.4	69.25	65.1	113	174.5	78.99
PLA/NT-N_2_-TMCTS	59.46	93.03	178	79.93	62.0	96	179	83.17

**Table 4:** Dynamic Mechanical Properties of PLA and PLA-based Composites.

Sample	(Tg)	(E′)	(E″)	Tanδ at Tg
PLA	68.65	3428	236	70.43
PLA/NT	59.25	6468	498	69.24
PLA/NT-N_2_	64.77	6729	1226	65.76
PLA/NT-N_2_-TMCTS	61.66	8051	1464	64.77

## Data Availability

Dataset available on request from the authors.
